# Authentification of fruit spirits using HS-SPME/GC-FID and OPLS methods

**DOI:** 10.1038/s41598-020-75939-0

**Published:** 2020-11-03

**Authors:** Tomáš Bajer, Martin Hill, Karel Ventura, Petra Bajerová

**Affiliations:** 1 Department of Analytical Chemistry, Faculty of Chemical Technology, University of Pardubice, Studentská 573, 532 10 Pardubice, Czech Republic; 2 Institute of Endocrinology, Národní 8, 116 94 Prague, Czech Republic

**Keywords:** Analytical chemistry, Cheminformatics, Statistics

## Abstract

This research provides an accurate description of the origin for fruit spirits. In total, 63 samples of various kinds of fruit spirits (especially from apples, pears, plums, apricots and mirabelle) were analysed using headspace-solid phase microextraction and gas chromatography with flame-ionization detector. Obtained volatile profiles were treated and analysed by multivariate regression with a reduction of dimensionality-orthogonal projections to latent structure for the classification of fruit spirits according to their fruit of origin. Basic result of statistical analysis was the differentiation of spirits to groups with respect to fruit kind. Tested kinds of fruit spirits were strictly separated from each other. The selection was achieved with a specificity of 1.000 and a sensitivity of 1.000 for each kind of spirit. The statistical model was verified by an external validation. Hierarchical cluster analysis (calculation of distances by Ward’s method) showed a similarity of volatile profiles of pome fruit spirits (apple and pear brandies) and stone fruit spirits (especially mirabelle and plum brandies).

## Introduction

Fruit spirits (FS) are known around the world due to their taste and aroma. Their production has a long tradition among Central European countries and many nations have them as a traditional alcoholic beverage.

Fruit spirits can be produced from fruits with high amounts of sugar, e.g. cherries, apples, pears, apricots, plums, peaches, mirabelles or raspberries. Fruit (crushed or sliced) or fruit juice is subjected to alcoholic fermentation, followed by double distillation and the treatment of final distillate (dilution, filtration etc.). FS are typically colourless (except spirits stored in wood barrels) with a specific fruit fragrance.

Fruit spirits contain ethanol, water and many other volatile substances (like higher alcohols, carbonyl compounds, esters, acids, terpenes, volatile phenols etc.) forming volatile profile, which plays the main role in the organoleptic characteristic of the spirits. The chemical composition of volatile profile depends on many factors like the type of fruit and its quality, conditions of the alcoholic fermentation and storage conditions. An important factor is also the aging of fruit spirits, which usually lasts several months to several years, but also has limits for some types of spirits. The composition of a volatile profile consists of volatile organic compounds (VOC) originating from fruit, volatile by-products formed during an alcoholic fermentation, and compounds formed during aging due to various chemical reactions. The taste of spirits can be affected by their storage in wooden barrels.

There are a number of studies found on the compositions of volatile compounds in fruit spirits, such as plum brandy^1,2^, pear brandy^3^, apricot brandy^4^, apple brandy^5^, mirabelle brandy^6^ or raspberry brandy^7^. Some of them also dealt with the comparison of aromatic compounds in different kinds of fruit spirits^5,8^. Characteristic compounds for FS are esters (typically ethylesters such as ethyl caprylate, ethyl caprate, ethyl laurate, ethyl myristate, ethyl palmitate and others) which are common for many types of FS and form dominant part of aroma profile^7^. Differences between different types of distillates are caused by other volatile substances that are part of a complex matrix containing hundreds of volatile compounds. For the analysis of chemical composition of volatile compounds some suitable extraction technique must be used. But, even with the use of appropriate extraction techniques in connection with gas chromatography-mass spectrometry, it is not easy to identify so many compounds to comparing the volatile profiles of each fruit distillate. Many substances that may be crucial for such analysis will remain unidentified and may remain outside the comparison of each type of distillate.

The aim of this research was to analyse VOC profiles of various FS (plum, mirabelle, apricot, pear and apple spirits) by method of headspace solid-phase microextraction coupled to gas chromatography with flame-ionization detection (HS-SPME/GC-FID) and obtained chromatographic data used for authentication of FS. HS-SPME/GC-FID was chosen as a screening procedure to classify complex chemical mixtures, such as fruit spirits samples and as a low-cost analytical technique which allowed measuring proper fingerprints based on composition of volatile compounds emitted from fruit spirits. Classification models were built to understand whether volatile compounds can be effectively used to discriminate fruit spirits according to their origin. In addition, origin identification of fruit spirits is also an interesting topic, due to the different price of distillates according to their fruit origin.

To interpret the relationships between volatile profiles and kind of fruit spirit, the method of multivariate regression with reduction of dimensionality—orthogonal projections to latent structures (OPLS) was used. This method based on the procedure of partial least squares (PLS) by using non-linear iterative partial least squares (NIPALS). OPLS method was proposed by Trygg and Wold^9^ and it is the modification of NIPALS PLS algorithm. Its objective is to improve the interpretation of PLS models by separating the systematic variation from an input dataset X (matrix with predictors and subjects) not correlated to the response set Y (matrix with dependent variables and subjects).

Moreover, OPLS method was used for the purpose of discriminant analysis (OPLS-DA)^10^. It was for investigating the similarity of volatile profiles of tested fruit spirits by hierarchical cluster analysis (HCA). Cluster analysis is a method of dealing with the similarity of multidimensional objects that it classified into clusters based on their similarity. It is used especially where the set of objects itself breaks down into classes, i.e. objects are naturally grouped into clusters.

## Results and discussion

### Classification analysis

**Figure 1:**
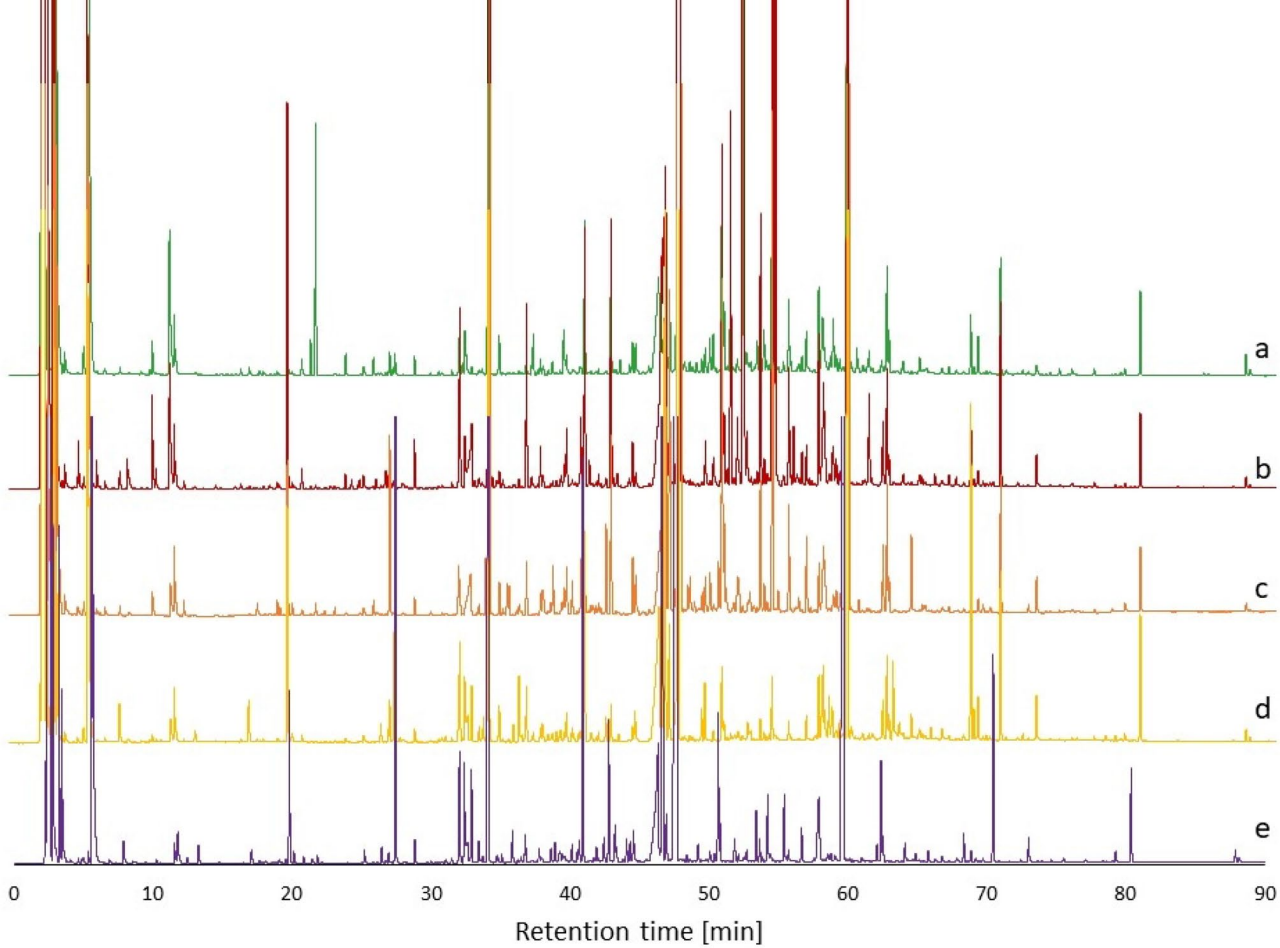
Typical chromatograms of volatile profiles of different types fruit spirits—a = pear spirit; b = apple spirit; c = apricot spirit, d = mirabelle spirit; e = plum spirit.

Before statistical analysis of measured data, peaks in individual GC-FID chromatograms were integrated and chromatographic data (peaks) was expressed as retention indices with the appropriate peak area. Retention indices were calculated using van Den Dool and Kratz formula^11^. Typical chromatograms of volatile profiles of different types fruit spirits are depicted in Fig. 1. All the samples were divided into two groups. The first group was a training set of 47 objects (8 pear spirits, 13 apple spirits, 5 apricot spirits, 8 mirabelle spirits, 13 plum spirits) and it was used for calibration of the model. The second group of 16 samples (2 plum spirits, 2 apple spirits, 1 pear spirit and 11 spirits labelled as “others”— “Samples” in “Materials and methods” section) was used as a test set for external validation.

### Creation of calibration model

Chromatographic data was converted to a matrix X (r × c) containing r lines (fruit spirit samples) and c columns (calculated retention indices of peaks in chromatograms with individual peak areas). Dataset was autoscaled (unit variance scaling). The logarithm of the ratio by the probability that the subject belongs to specific class of fruit spirit to the probability that the subject is of another class (logarithm of the likelihood ratio, LLR) was chosen as a dependent variable Y, while individual peaks, labelled by the value of retention indices with peak areas, were the predictors. The actual LLR values were calculated according to Eq. (1). Actual LLR values are theoretically equal to – ∞ or + ∞, but positive or negative numerical values have been used for practical evaluation. All objects of investigated class of fruit spirits have a positive value of LLR, all objects which don´t belong to an investigated class of fruit spirits have a negative value of LLR. In this case, the class threshold is given as a value of LLR = 0.

1 $$\text{LLR } = \ln\left(\frac{\text{A}}{{\text{B}}}\right)$$

A—probability that subject belong to a specific category, B—probability that subject does not belong to a specific category.

First, measured data was transformed to obtain a symmetric distribution. For the separation of uncorrelated information, two statistics were used:

1. Multivariate t-test (Hotelling’s T2 statistic) for checking the homogeneity in predictors; T2 values represent the distance from the origin in the model plane (score space) for each observation. Values larger than the 95% confidence limit are suspect, and values larger than the 99% confidence limit can be considered as serious (manual of SIMCA software). T2 value far above the critical limits indicates that the observation is far from the other observations in the selected range of components for the score space. In this case observation is considered as outlier that, if in the training set, may pull the model in a detrimental way.
2. Variable importance for the projection (VIP) for testing of relevance of predictors; VIP values represent the importance of the variables both to explain X and to correlate to Y. Relevant X-variables are indicated by values larger than 1, and values lower than 0.5 indicate “unimportant” X-variables. The rest of the variables have a VIP value in the interval between 0.5 and 1, their importance depends on the size of the dataset (manual of SIMCA software). The criterion of VIP value > 1 is often use as a cut-off point for variable selection^12^. For example, it was used as a cut-off point for selection of relevant variables in work of Giannetti^13^. Nevertheless, data structures are diverse and may vary from case to case, and therefore the cut-off point may not be the same for different data structures. Determining the appropriate cut-off point is not simple, because extremely high values can remove key variables and extremely low values can include variables that are not relevant to the analysis^12^. In our work variables of VIP values of 95% confidence limit were not considered.

After separation of uncorrelated information; the following parameters were calculated: (a) component loadings for individual variables to evaluate relationships between predictors and kind of distillate for each predictive components, (b) regression coefficients for the multiple regression model to evaluate relationships between predicted variables (LLR) and predictors (it is a specific predictor effect that does not depend on the predictors remaining), (c) predicted values of the LLR, (d) sensitivity and specificity of the prediction.

Using the procedures described above an OPLS model was obtained which was characterized by six components. Four of them were predictive components. These components expressed information found in X and Y, i.e. they summarize information in X that is predictive to Y. The remaining two components were orthogonal and expressed information found only in X, i.e. they express systematic information that is unique in X (orthogonal in Y). In Table 1, there are components loadings of predicted variable for each predictive component together with correlation coefficients and explained variability. Model explained 92.7% of variation (R2) of the training set explained by the predictive components. It is a measure of suitability, i.e. how well the model fits the data. Predicted variation (Q2) of the training set according to cross validation is 78.0%. It indicates that the model predicts new data well. The best fruit spirits differentiation within one component was achieved with the maximum possible difference in correlation coefficients. Diverse kinds of fruit spirit separation were done by each component. Pome fruit (pears and apples) spirits was separated from plums by the 1st predictive component P1. Difference between apple and pear distillates was given mainly by the 4th predictive component P4. Differences inside the group of stone fruit spirits were given by various predictive components; components P1 and P2 separate plum spirits from other stone fruit spirits, the combination of P4, P3 and P2 separates mirabelle spirits, and P3 separates apricot spirits. This OPLS model was applied on the training set and it provided a correct classification for all five classes of distillates based on sensitivity and specificity 1.000.

**Table 1:** Relationships between the kind of fruit distillates and predictive variables for each predictive component as evaluated by OPLS model.

Predictive component	Predictive variable^a^	Component loadings	t-statistic	Correlation coefficient	Explained variation, R2 (predicted variation, Q2)
P1	PEA_LLR	0.086	6.71	0.562*	25.6% (20.1%)
	APP_LLR	0.086	5.51	0.542*	
	APR_LLR	− 0.026	− 1.60	− 0.177	
	MIR_LLR	− 0.024	− 1.01	− 0.130	
	PLU_LLR	− 0.120	− 7.83	− 0.783*	
P2	PEA_LLR	0.017	0.51	0.086	23.4% (13.3%)
	APP_LLR	− 0.093	− 2.08	− 0.441*	
	APR_LLR	0.071	0.70	0.382	
	MIR_LLR	0.155	1.51	0.746	
	PLU_LLR	− 0.100	− 4.21	− 0.521*	
P3	PEA_LLR	− 0.047	− 1.43	− 0.240	21.5% (24.5%)
	APP_LLR	0.041	0.61	0.174	
	APR_LLR	0.173	2.07	0.870*	
	MIR_LLR	− 0.094	− 1.10	− 0.432	
	PLU_LLR	− 0.041	− 0.61	− 0.209	
P4	PEA_LLR	0.187	8.57	0.731*	22.2% (20.1%)
	APP_LLR	− 0.157	− 9.62	− 0.617*	
	APR_LLR	0.040	0.88	0.157	
	MIR_LLR	− 0.093	− 3.74	− 0.368*	
	PLU_LLR	0.051	2.93	0.204*	
Total explained variability by OPLS model					92.7% (78.0%)

*p < 0.01.

^a^LLR—logarithm of the likelihood ratio (logarithm of the ratio of the probability that the subject belongs to specific class of fruit distillate to the probability that the subject is of other class), PEA—pear, APP—apple, APR—apricot, MIR—mirabelle, PLU—plum.

### External validation of the models

**Figure 2:**
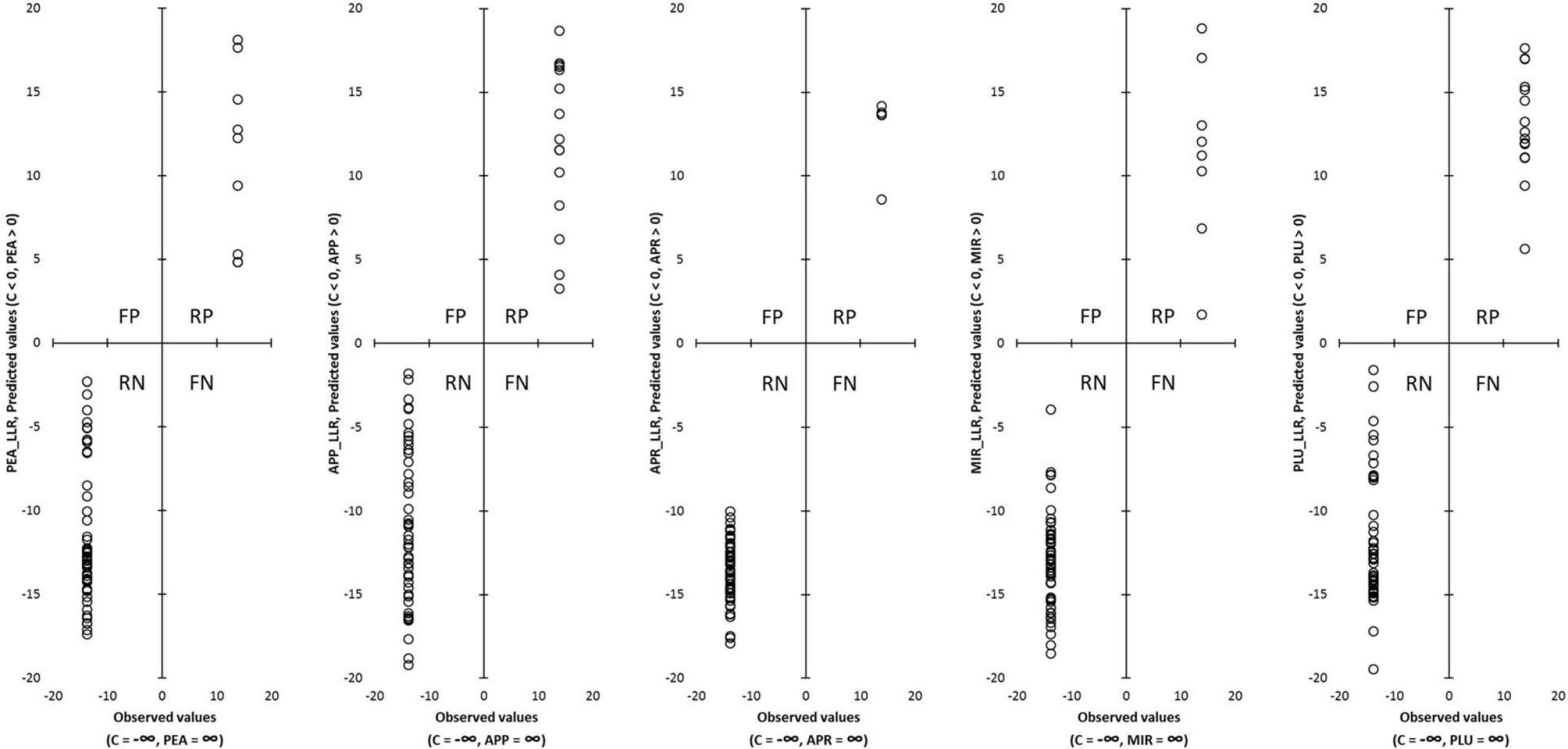
Discrimination of fruit spirit samples according type of fruit on the basis of volatile compounds fingerprint obtained by HS-SPME/GC-FID and using OPLS. LLR—logarithm of the ratio of the probability that the subject belongs to specific class of fruit distillate to the probability that the subject is a control (actual LLR (observed values) of given type of fruit spirit = ∞, actual LLR for controls =  − ∞). PEA—pear, APP—apple, APR—apricot, MIR—mirabelle, PLU—plum; FP—quadrant of false positive subjects, RP—quadrant of really positive subjects, RN—quadrant of really negative subjects, FN—quadrant of false negative subjects.

The aim of this work was to develop a method that would be applicable for the verification of spirits from apples, pears, mirabelles, plums and apricots, to determine whether the samples are spirits of a specified kind or not. For this purpose, the created OPLS model was applied to both datasets, training and testing (63 samples in total). The results were graphically plotted (Fig. 2) by the dependencies of observed (actual) LLR values against the LLR values calculated (predicted) by the OPLS model. The actual LLR values were calculated according to Eq. (1). All the tested subjects in the OPLS model were sequentially assigned to the appropriate quadrants of each graph. The results show, that all positive subjects (samples of given type of fruit distillate) were marked as positive, and none of the negative subjects were marked as false positive. According to these results, the model can predict if sample of the distillate is of the same type as is declared. These data show that chromatographic data (chromatographic peaks characterized by retention indices and by peak areas) obtained by the HS-SPME/GC-FID method can be used as an identification of single-fruit spirits to verify their authenticity, and that some volatiles and especially their combinations can play a key role in the controlling of authentication of fruit spirits. It also points to the selection of the correct extraction method parameters.

### Similarity of volatile profiles

**Figure 3:**
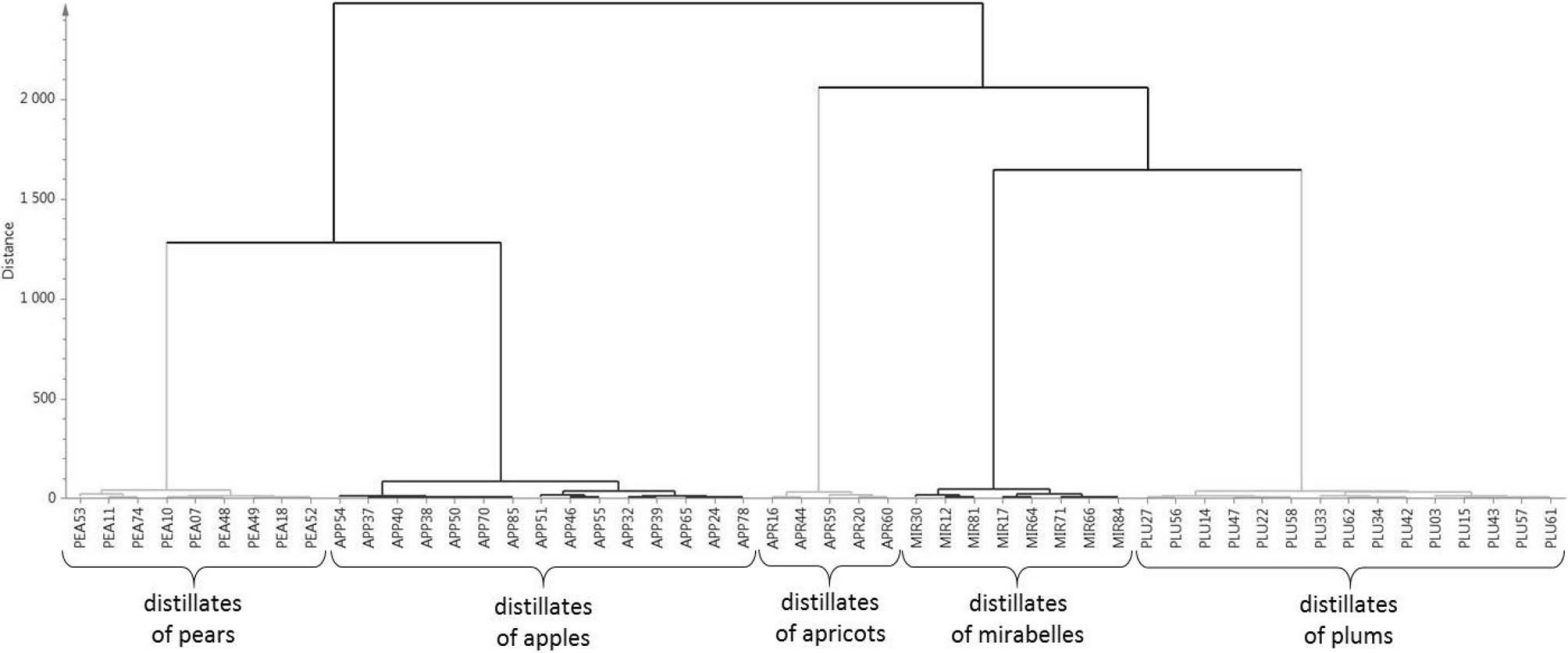
Dendrogram from hierarchical cluster analysis. Ward variance function was used as a linkage criterion.

This analysis was carried out as a supplement to obtain additional information on the similarity/dissimilarity for the volatile profiles of the different kinds of distillates. HCA was used for study of volatile profiles for all the tested spirits except “other distillates” (see Table 1) after OPLS-DA separated systematic variations in matrix X orthogonal to Y. The dendrogram from HCA is depicted in Fig. 3. The vertical scale (Euclidean distance) is a similarity measurement, with similarity increasing as the numerical value gets closer to zero. Thus, when distances between the observations are relatively small, it implies that the observations are similar to some degree. On the other hand, when distances between the observations are larger, it implies that the observations are markedly different^14^.

There are two clusters in the dendrogram, both of which are divided into sub-clusters. Cluster on the right side is formed by three sub-clusters. All three sub-clusters are formed by the samples of stone fruit spirits (plum, apricot and mirabelle spirits). Volatile profiles of all these spirits are similar. Location of plum and mirabelle clusters give the information that their volatile profiles are similar, and they differ from volatile profile of apricot spirits. The similarity of the volatile profiles of plum and mirabelle distillates is expected since both fruits are of the same species. The only difference is that mirabelle (*Prunus domestica* subsp. *syriaca*) is a subspecies of plum (*Prunus domestica*).

Cluster on the left side is made up of sub-clusters of pear and apple spirits, so it means that these two sub-clusters are similar. Apples (species *Malus*) and pears (species *Pyrus*) are pome fruits, so it is obvious, that their volatile profiles can be similar. Interestingly, the similarity of the volatile profiles of these spirits is higher than in case of volatile profiles of plum and mirabelle spirits.

## Conclusions

The aim of this research was to develop a method for the accurate determination of the origins of fruit spirits. Analysis of 63 samples were performed using headspace-solid phase microextraction and gas chromatography with flame-ionization detector. The obtained data was analysed by the method of orthogonal projections to latent structure. Statistical models were verified by external validation. The resulting distribution of all tested samples exactly corresponds to the type of individual distillates. Hierarchical cluster analysis provided information about the degree of similarity/dissimilarity between the tested kinds of distillates. The limitation of developed model is possible positive classification only for five types of fruit spirits (plum, apricot, apple, pear and mirabelle spirits). Samples of other types of distillates will not be assigned to any of that five types.

## Materials and methods

### Samples

A set of 63 samples of distillates were analysed in this study. All the distillates were produced in different parts of the Czech Republic. Different types of fruit for the making of distillates was grown in the Czech Republic as well. Samples were obtained from local growers and producers, who guaranteed their origin and authenticity. Distillates were made from plums (15 samples), apricots (5), apples (15), pears (9) and mirabelles (8), next 11 samples were made from other plant material (see Table 2).

**Table 2:** List of the samples of distillates.

Kind of spirit	Vintage	Label	Kind of spirit	Vintage	Label
**Distillates of apples (15)**			**Distillates of plums (15)**		
Moravian-Silesian region	2013	APP78	Moravian-Silesian region	2007	PLU27
Moravian-Silesian region	2014	APP24	Moravian-Silesian region	2007	PLU61
Moravian-Silesian region	2014	APP32	Moravian-Silesian region	2012	PLU14
Pardubice region	2014	APP50	Moravian-Silesian region	2013	PLU15
Moravian-Silesian region*	2015	APP37	Zlín region	2013	PLU58
Moravian-Silesian region	2015	APP38	Moravian-Silesian region	2014	PLU03
Moravian-Silesian region	2015	APP39	Moravian-Silesian region	2014	PLU22
Moravian-Silesian region	2015	APP40	Moravian-Silesian region	2014	PLU33
Moravian-Silesian region	2015	APP51	Moravian-Silesian Region	2014	PLU42
Hradec Králové region	2016	APP55	Vysočina region	2015	PLU47
Moravian-Silesian region*	2016	APP65	Hradec Králové region	2015	PLU56
Moravian-Silesian region	2016	APP70	Moravian-Silesian region	2015	PLU62
Central Bohemian region	2016	APP85	Moravian-Silesian region*	2016	PLU34
Vysočina Region	2017	APP46	Moravian-Silesian region	2016	PLU43
Hradec Králové region	2017	APP54	Hradec Králové region*	2016	PLU57
**Distillates of mirabelles (8)**			**Distillates of apricots (5)**		
Zlín region	2003	MIR71	Moravian-Silesian region	2011	APR20
Moravian-Silesian region	2012	MIR12	Zlín region	2013	APR16
Moravian-Silesian region	2013	MIR17	Zlín region	2014	APR44
Moravian-Silesian region	2014	MIR30	South Moravian region	2014	APR60
Moravian-Silesian region	2014	MIR81	Zlín region	2016	APR59
Moravian-Silesian region	2015	MIR64	**Other distillates (11)**		
Central Bohemian region	2015	MIR84	Walnut, Moravian-Silesian region*	2014	
Moravian-Silesian region	2017	MIR66	Elderberry, Moravian-Silesian region*	2014	
**Distillates of pears (9)**			Pumpkin, Moravian-Silesian region*	2014	
Moravian-Silesian region	2009	PEA10	Rye, Moravian-Silesian region*	2014	
Moravian-Silesian region	2012	PEA11	Raspberry, Moravian-Silesian region*	2015	
Moravian-Silesian region	2014	PEA07	Elderberry, Moravian-Silesian region*	2015	
Moravian-Silesian region	2014	PEA18	Elderflower, Moravian-Silesian region*	2015	
Moravian-Silesian region	2014	PEA52	Cherry, Moravian-Silesian region*	2015	
Moravian-Silesian region	2016	PEA74	Grapes, Moravian-Silesian region*	2015	
Vysočina region	2016	PEA48	Cherry, South Moravian region*	2016	
Pardubice region*	2017	PEA49	Grapes, South Moravian region*	2017	
Hradec Králové region	2017	PEA53			

*Chromatographic data were used in external validation dataset.

### Chemicals and materials

The *n*-alkane standard solution of C8–C33 was purchased from CPAChem (Stara Zagora, Bulgaria). Distilled water was purified using a Milli-Q system from Merck (Darmstadt, Germany). Sodium chloride (analytical grade) was purchased from Lach-Ner s.r.o. (Neratovice, Czech Republic). The SPME fiber 100 μm PDMS (polydimethylsiloxane) was purchased from Supelco (Bellefonte, PA, USA).

### Headspace solid-phase microextraction method

Experimental conditions used for the headspace solid-phase microextraction (HS-SPME) method were the same as those previously described^7^. Briefly, they were as follow: 2 mL of fruit spirit and 8 mL of sodium chloride water solution (28.5% (w/v)) were transferred into a 20 mL vial and properly mixed. The vial was closed using a cap with Teflon septum. Samples were pre-incubated at 45 °C for 20 min to ensure steady-state extraction conditions. The extraction of volatile compounds was performed using 100 μm PDMS fiber at 45 °C for 60 min. Then, the extracted volatile compounds were automatically desorbed into the GC injection port at 200 °C.

### Chromatographic analysis

A gas chromatograph (GC), model GC-2010 Plus, with a flame-ionization detector (FID) from Shimadzu (Kyoto, Japan) was used for the separation of volatile compounds. A Combi PAL autosampler (CTC Analytics, Zwingen, Switzerland) equipped with an agitating and heating unit was used for the automated HS-SPME procedure, including desorption of analytes from the fiber into an injector as well as cleaning of the fiber in a cleaner system after desorption. SLB-5 ms (Supelco, Bellefonte, PA, USA) capillary column (30 m × 0.25 mm; 0.25 μm of film thickness) was used for separation. Helium 5.0 (Linde Gas a.s., Prague, Czech Republic) was used as a carrier gas at a constant linear velocity of 30 cm/s. The injector was maintained at 200 °C and desorption time was 15 s. Injections were carried out in a split mode and split ratio of 1:20. The column temperature program was set up as follow: the initial temperature was held at 40 °C for 3 min and then increased up to 250 °C by 2 °C/min, held for 12 min. Temperature of the flame-ionization detector was set to 270 °C. The hydrogen flow rate in a detector was 40 mL/min and air flow rate was 400 mL/min. Nitrogen was used as a make-up gas with a flow rate of 30 mL/min. Mixture of standard *n*-alkanes (C8-C33) was analysed at the same chromatographic conditions as the samples^7^. *n*-Alkanes solution was measured for calculation of retention indexes (RI).

### Data analysis

Statistical analysis was done using statistical software SIMCA version 13.0 (Sartorius Stedim Data Analytics AB, Umeå, Sweden). The main analysis, classification analysis, was performed by OPLS. Then, as a minor analysis, testing of similarity of volatile profiles was performed by HCA.

### Determination of kind of fruit spirit

Classification of fruit spirits were done by the evaluation of relationships between chromatographic data and the kinds of fruit spirits by multivariate regression with a reduction of dimensionality known as OPLS. This method is able to deal with the problem of multicollinearity (high mutual correlation) in the matrix of predictors, while normal multiple regression does not evaluate such data; thus, increasing the model’s predictability.

The OPLS model is an extension of the PLS model. It separates the systematic variation of X into two parts, one that is predictive to Y and one that is orthogonal to Y. The X/Y predictive variation is modelled by the predictive components. The variation in X which is orthogonal to Y is modelled by the orthogonal components. Variable importance in projection (VIP) statistics were used to select predictors relevant to the analysis. This method is useful for determining which characteristics have contributed most to class separation.

### Testing of similarity of volatile profiles

HCA was used to determine the similarity/dissimilarity of the volatile profiles belonging to different types of distillates in terms of fruit type. HCA is most often used for the clustering of observations and may play a central role in the definition of new classes, as well as corroborating the existence of classes that have been detected by PCA or perhaps are anticipated based on prior knowledge or experience. From the viewpoint of cluster recognition, one may therefore regard HCA as an explanatory tool. Cluster analysis can be used to discover structures in data without the need for an explanation/interpretation. The outcome of HCA depends on the distance metric used and the linkage criterion that is selected^14^. The SIMCA software only works by considering Euclidean distances, there is a choice between the linkage function due to Ward and the single linkage approach. The variance function of Ward was used as the criterion for choosing the pair of clusters to merge. It calculates the difference in the sum of squares around mean of each cluster before and after merging two clusters. For the HCA plot, the vertical axis indicates the loss in cluster similarity, i.e., the variance increases, when clusters are merged.
